# Statin and rottlerin small-molecule inhibitors restrict colon cancer progression and metastasis via MACC1

**DOI:** 10.1371/journal.pbio.2000784

**Published:** 2017-06-01

**Authors:** Manisha Juneja, Dennis Kobelt, Wolfgang Walther, Cynthia Voss, Janice Smith, Edgar Specker, Martin Neuenschwander, Björn-Oliver Gohlke, Mathias Dahlmann, Silke Radetzki, Robert Preissner, Jens Peter von Kries, Peter Michael Schlag, Ulrike Stein

**Affiliations:** 1 Experimental and Clinical Research Center, Charité - Universitätsmedizin Berlin and Max-Delbrück-Center for Molecular Medicine, Berlin, Germany; 2 Leibniz-Institut für Molekulare Pharmakologie (FMP), Berlin, Germany; 3 Charité - University Medicine Berlin, Structural Bioinformatics Group, Institute of Physiology & Experimental Clinical Research Center, Berlin, Germany; 4 German Cancer Consortium (DKTK), Heidelberg, Germany; 5 Charité Comprehensive Cancer Center, Berlin, Germany; Stanford University School of Medicine, United States of America

## Abstract

MACC1 (Metastasis Associated in Colon Cancer 1) is a key driver and prognostic biomarker for cancer progression and metastasis in a large variety of solid tumor types, particularly colorectal cancer (CRC). However, no MACC1 inhibitors have been identified yet. Therefore, we aimed to target MACC1 expression using a luciferase reporter-based high-throughput screening with the ChemBioNet library of more than 30,000 compounds. The small molecules lovastatin and rottlerin emerged as the most potent MACC1 transcriptional inhibitors. They remarkably inhibited MACC1 promoter activity and expression, resulting in reduced cell motility. Lovastatin impaired the binding of the transcription factors c-Jun and Sp1 to the MACC1 promoter, thereby inhibiting MACC1 transcription. Most importantly, in CRC-xenografted mice, lovastatin and rottlerin restricted MACC1 expression and liver metastasis. This is—to the best of our knowledge—the first identification of inhibitors restricting cancer progression and metastasis via the novel target MACC1. This drug repositioning might be of therapeutic value for CRC patients.

## Author summary

Cancer is still one of the leading causes of death in the Western world, and metastasis—the spread of cancer to distant sites—represents the most critical attribute for therapy failure. In colorectal cancer, up to one-third of patients have already developed metastasis at the time of diagnosis, and about half of newly diagnosed patients will develop metastasis during the course of the disease. MACC1 was first described as a key driver of metastasis formation in colorectal cancer, and its importance was later confirmed for other solid tumor entities. Stratification of patients with high MACC1 expression identifies patients at high risk of developing metastasis. Here we present a mechanism of targeting MACC1 as a potential therapy option for these high-risk patients. We identify the small molecules lovastatin and rottlerin as transcriptional inhibitors of MACC1. We describe the mechanism by which these molecules inhibit MACC1 expression and show that MACC1 inhibition leads to a reduced migratory phenotype in vitro and limits metastatic spread in preclinical mouse models. We propose repositioning of these 2 known drug molecules to reduce MACC1-driven metastasis formation in high-risk patients even before metastatic spread is clinically evident.

## Introduction

Colorectal cancer (CRC) is one of the leading causes of cancer-associated death worldwide. Metastasis of CRC is mainly responsible for the global mortality burden and is directly linked to patient survival. This necessitates the search for molecular biomarkers for the early identification of patients with tumors of elevated metastatic propensity. One such promising biomarker that emerged in the recent past is Metastasis-Associated in Colon Cancer 1 (MACC1) [1,2]. MACC1 mRNA expression in primary tumors was shown to be directly correlated with metastasis formation and metastasis-free survival within a 12-y follow-up [1]. Numerous follow-up studies confirmed the prognostic value of MACC1 for CRC metastasis and patient survival [3–9]. MACC1 was shown to induce migration, invasion, and proliferation in cell culture, as well as tumor progression and formation of metastases in xenografted and genetically engineered mouse models [1,10,11]. Many further studies reported that MACC1 can act as a decisive driver for the transition from adenoma to carcinoma and thus initiates cancer progression and ultimately metastasis [5,10,12–18]. Apart from its crucial role in CRC progression and metastasis, recent studies indicate the relevance of MACC1 in tumor progression and metastasis of several other solid tumor types [2,3,12,17,19–26]. All these studies have speculated about the strong therapeutic potential of targeting MACC1 to restrict CRC progression and metastasis, which can also be applied to other solid cancers. However, so far, no inhibitor of MACC1 expression has been described.

Here we report the identification of the first small-molecule MACC1 transcriptional inhibitors using a high-throughput screening (HTS) of more than 30,000 compounds, which was possible because of our previous identification of the human MACC1 promoter [27]. We identified the statins mevastatin and lovastatin and, additionally, rottlerin as effective inhibitors of MACC1 promoter activity and expression. Statins are originally a widely known and clinically used drug class for reducing cholesterol levels [28]. Rottlerin shows different modes of action but has not been successful in achieving clinical approval [29].

In this study, we particularly investigated the effects of lovastatin on MACC1 expression and MACC1-associated metastasis in order to reposition this known compound as an antimetastatic drug, thereby broadening its therapeutic value in oncology.

## Results

### HTS led to identification of mevastatin and rottlerin as transcriptional inhibitors of MACC1

**Fig 1:**
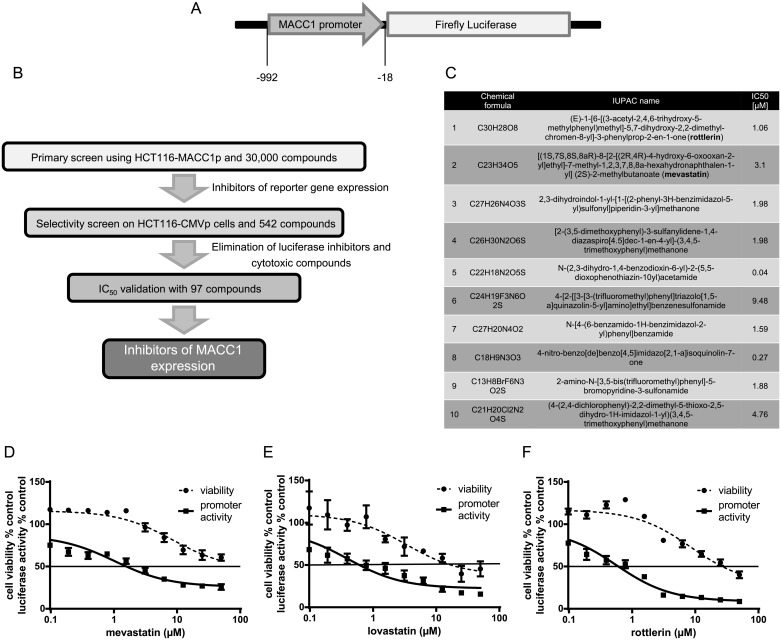
Identification of the MACC1 transcriptional inhibitors via high-throughput screening (HTS). (A) Schematic representation of the reporter system used in the screening. The expression of the reporter firefly luciferase was regulated by the human MACC1 promoter (−992 to −18 bp upstream of the MACC1 transcriptional start site). (B) Diagrammatic representation of the HTS of HCT116-MACC1p-Luc cells with MACC1 promoter-driven luciferase expression. (C) Top 10 identified MACC1 promoter inhibitors identified from the HTS. (D–F) HCT116-MACC1p-Luc cells were treated with 10 two-fold serial dilutions of mevastatin (D), lovastatin (E), and rottlerin (F) for 24 h, starting with a 25 μM drug concentration. Luciferase activity was determined using steady glow luciferase reagent and normalized to untreated cells. Cell viability was measured independently by MTT assay. Results are shown as mean ± SEM of at least 2 independent experiments performed in triplicate.

HCT116-MACC1p-Luc CRC cells stably expressing the human MACC1 promoter-driven luciferase reporter gene (Fig 1A) were used to screen the ChemBioNet library of more than 30,000 compounds, which includes the Sigma Library of Pharmacologically Active Compounds (LOPAC), for identification of potential transcriptional MACC1 inhibitors [30]. In the primary screen, using 5 μM of each compound (the screening parameters are listed in S1 Table), we identified 542 compounds that inhibited MACC1 promoter-driven luciferase expression by more than 3 standard deviations from the mean of all samples on a plate (Z score < −3; Fig 1B). These 542 compounds were then subjected to a selectivity counter screen for false positives acting against luciferase itself with HCT116-CMVp-Luc cells, wherein the luciferase gene was driven by the CMV promoter instead of the MACC1 promoter. Four hundred and forty-five compounds inhibited CMVp-driven luciferase expression by more than 75%; these were considered as nonspecific luciferase inhibitors, CMVp inhibitors, or cytotoxic compounds and were excluded. Ninety-seven specific compounds, which included 7 pharmacologically active compounds from the LOPAC of approved drugs and 90 novel biologically annotated compounds, were left for further characterization. These 97 compounds were then tested for MACC1p inhibitory capacity in more detail by luciferase assays using 10 two-fold serial dilutions starting with the highest concentration of 25 μM. On the basis of inhibitory properties (Hill coefficient and IC_50_ values), solubility, purity, selectivity screen comparison, and information on known biological targets and functions, the top 10 candidates with the most potential were identified (Fig 1C, S2 Table). Among these 10 compounds, mevastatin and rottlerin showed the best potential for MACC1 transcriptional inhibition. In the dose-response assay for measuring MACC1 promoter activity, mevastatin and rottlerin showed a remarkable inhibition of luciferase activity at a concentration of 1.6 μM and 0.78 μM, respectively (Fig 1D and 1F). We next performed an MTT assay to evaluate the effects of these drugs on cell viability. Mevastatin and rottlerin reduced cell viability by 50% at concentrations higher than 50 and 25 μM, respectively (Fig 1D and 1F).

However, in the past mevastatin was evaluated as a less effective HMG-CoA inhibitor in patients and demonstrated an acute toxicity profile in dogs [31]. Because of these controversial findings, mevastatin did not reach routine clinical use. In contrast, lovastatin exerted better efficacy as an HMG-CoA inhibitor and became the first member of the statins receiving Food and Drug Administration (FDA) approval [28]. Moreover, mevastatin and lovastatin are structurally closely related compounds that differ only by 1 methyl group. Although lovastatin was not included in the compound library, we decided to work with lovastatin from here on instead of mevastatin.

Therefore, we determined whether lovastatin has the same inhibitory effect on MACC1 promoter-driven reporter gene expression as mevastatin, allowing fast translation into clinical trials. We performed a dose-response curve using 10 two-fold serial dilutions of lovastatin as done for mevastatin and analyzed its effect on cell viability and luciferase activity in HCT116-MACC1p-Luc cells. Lovastatin inhibited luciferase activity at 0.39 μM and higher concentrations and reduced viability only at 12.5 μM and higher concentrations (Fig 1E).

Thus, the dose-response assays confirmed that mevastatin, lovastatin, and rottlerin possess the potential to inhibit MACC1 promoter-driven reporter gene expression at noncytotoxic concentrations.

### Mevastatin, lovastatin, and rottlerin inhibit MACC1 mRNA and protein expression in a time- and concentration-dependent manner

**Fig 2:**
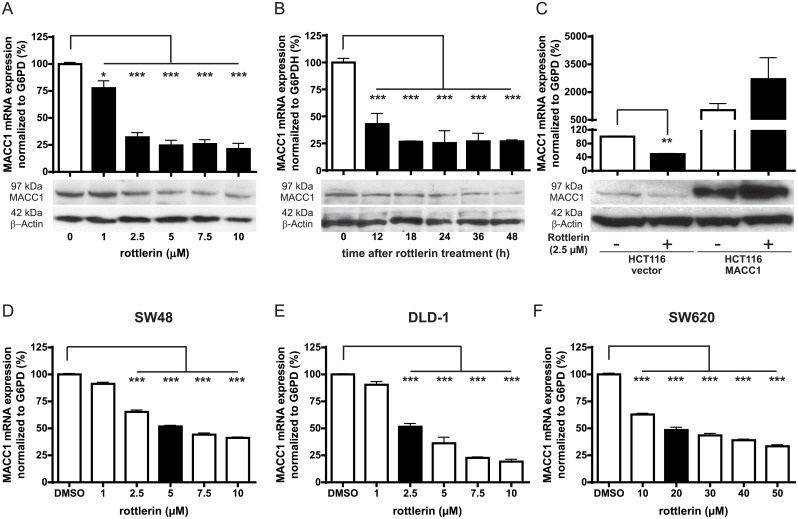
The effect of rottlerin on MACC1 expression. (A–B) HCT116 cells were treated with increasing concentrations of rottlerin for 24 h (A) or a single dose of 2.5 μM rottlerin (B) for the time points indicated. MACC1 mRNA expression and protein expression were determined by quantitative real-time reverse-transcription polymerase chain reaction (qRT-PCR) and western blot analysis, respectively. Treated samples are shown with black bars. (C) HCT116/vector and HCT116/MACC1 cells were treated with 2.5 μM rottlerin for 24 h, and MACC1 mRNA and protein levels were analyzed (*p* < 0.01). (D–F) SW48 (D), DLD-1 (E), and SW620 (F) cells were treated with increasing concentrations of rottlerin for 24 h. MACC1 mRNA expression was analyzed by qRT-PCR. Samples with a 50% decrease in MACC1 mRNA levels are highlighted with black bars. MACC1 mRNA was normalized with G6PD and expressed as a percentage of solvent-treated cells (*p* < 0.001), whereas β-actin was used as loading control for western blotting. Data represent mean ± SEM (*n* ≥ 2), *p < 0.05, **p < 0.01 ***p < 0.001.

**Fig 3:**
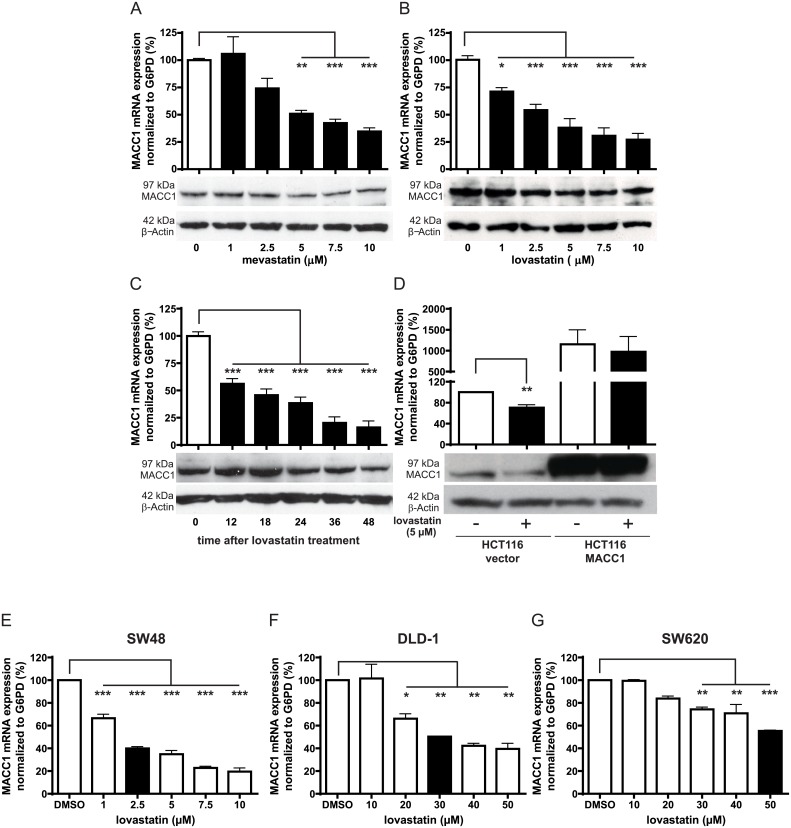
The effect of mevastatin and lovastatin on MACC1 expression. (A–B) HCT116 cells were treated with increasing concentrations of mevastatin (*p* < 0.01, *p* < 0.001) (A) and lovastatin *(p* < 0.05, *p* < 0.001) (B) for 24 h. MACC1 mRNA expression and protein expression were determined by quantitative real-time reverse-transcription polymerase chain reaction (qRT-PCR) and western blot analysis, respectively. Treated samples are shown with black bars. MACC1 mRNA was normalized with G6PD and expressed as a percentage of solvent-treated cells. (C) HCT116 cells were treated with a single dose of 5 μM lovastatin for the time points indicated. MACC1 mRNA expression and protein expression were determined by qRT-PCR and western blot analysis, respectively (*p* < 0.001). (D) HCT116/vector and HCT116/MACC1 cells were treated with 5 μM lovastatin for 24 h, and MACC1 mRNA and protein levels were analyzed (*p* < 0.01). (E–G) SW48 (*p* < 0.001) (E), DLD-1 (*p* < 0.05, *p* < 0.01) (F), and SW620 (*p* < 0.01, *p* < 0.001) (G) cells were treated with 5 μM lovastatin for 24 h. MACC1 mRNA was analyzed by qRT-PCR, normalized with G6PD, and expressed as a percentage of solvent-treated cells. β-actin was used as the loading control for western blotting. Data represent mean ± SEM (*n* ≥ 2), *p < 0.05, **p < 0.01, ***p < 0.001.

To determine the effect of the identified inhibitors on MACC1 expression, HCT116 cells were treated with increasing concentrations of rottlerin, mevastatin, and lovastatin for 24 h. At a concentration of 2.5 μM, rottlerin showed more than 60% reduction in the endogenous MACC1 mRNA (*p* < 0.001) and protein level compared to the solvent-treated control (Fig 2A). Similarly, treatment with 5 μM mevastatin significantly restricted the MACC1 mRNA level to 50% of the solvent-treated control (Fig 3A, *p* < 0.01), whereas treatment with 5 μM lovastatin significantly reduced MACC1 mRNA levels by 62% as compared to the solvent-treated control (*p* < 0.001, Fig 3B). Consistent with the reduction of MACC1 mRNA levels, the statins caused a decrease in MACC1 protein level at 5 μM and above, as demonstrated by western blotting (Fig 3A and 3B). These results demonstrate that the treatment reduced the MACC1 mRNA level in a concentration-dependent manner for all 3 drugs.

Of note, we saw comparable efficiency of mevastatin and lovastatin as MACC1 transcriptional inhibitors.

Based on our primary interest of repositioning approved drugs as MACC1 inhibitors, lovastatin became the preferred drug candidate for our further, more detailed analyses.

We then analyzed the kinetics underlying the rottlerin- and lovastatin-mediated inhibition of MACC1 expression. We found that both drugs reduced the MACC1 expression after a single drug application. After 12 h of treatment of HCT116 cells with 2.5 μM rottlerin, MACC1 mRNA was significantly reduced to less than 50% (*p* < 0.001) as compared to the solvent-treated control and was further reduced to 26% of the solvent-treated cells (*p* < 0.001) at 48 h (Fig 2B). Consistent with the mRNA expression, reduction in the MACC1 protein was also detected 12 h post treatment and remained low until 48 h following a single dose (Fig 2B). For lovastatin, MACC1 mRNA was significantly reduced to less than 50% (*p* < 0.001) after 18 h of drug treatment with 5 μM lovastatin and was further decreased to 16% at 48 h (*p* < 0.001) as compared to the solvent-treated control (Fig 3C). Consistent with the mRNA expression, reduction in the MACC1 protein was also detected at 24 h post lovastatin treatment, and it remained reduced until 48 h following the single drug application (Fig 3C).

To further support that rottlerin and lovastatin are transcriptional inhibitors of MACC1, we hypothesized that ectopic overexpression of MACC1 governed by a promoter other than the human MACC1 promoter should be irresponsive to the inhibitory effects of lovastatin. Therefore, HCT116 cells were transiently transfected to express CMV promoter-driven MACC1 cDNA, leading to increased MACC1 mRNA and protein levels compared to HCT116/vector cells. Treatment of HCT116/vector cells with 2.5 μM rottlerin and 5 μM lovastatin (Figs 2C and 3D) reduced the MACC1 mRNA levels to more than 50% of the solvent-treated control (*p* < 0.01), which was similar to the effect observed in wild-type HCT116 cells. In contrast, treatment of HCT116/MACC1 cells with lovastatin did not result in inhibition of MACC1 mRNA. Coherent to the mRNA data, MACC1 protein expression in the HCT116/vector cells was reduced upon drug treatments, but not in drug-treated HCT116/MACC1 cells (Figs 2C and 3D).

### Lovastatin and rottlerin restrict MACC1 mRNA in a panel of CRC cell lines

Next, we analyzed the inhibitory effects of rottlerin and lovastatin on the MACC1 expression in the SW48, DLD-1, and SW620 CRC cell lines. Both drugs were capable of restricting MACC1 expression in these 3 human CRC cell lines. Since the effective drug concentrations were cell line dependent, we tested all drug concentrations for effects on cell viability. None of these concentrations including elevated drug concentrations of above 10 μM reduced cell viability within 24 h (S1 Fig). Upon treatment of these CRC cells with rottlerin, MACC1 mRNA levels were reduced to 50% at a concentration of 5 μM in SW48 cells, 2.5 μM in DLD-1 cells, and 20 μM in SW620 cells as compared to the solvent-treated control (*p* < 0.001, Fig 2D, 2E and 2F).

For lovastatin, treatments of 2.5 μM were sufficient to reduce MACC1 mRNA to 40% in SW48 cells, (*p* < 0.001, Fig 3E), whereas in DLD-1 cells, 30 μM of lovastatin was required to achieve a 50% decrease in MACC1 mRNA level (*p* < 0.01, Fig 3F). For SW620 cells, a higher concentration of 50 μM was required to restrict MACC1 expression to 55% (*p* < 0.001, Fig 3G).

However, to identify a potential MACC1 inhibitor with a brighter clinical translational perspective anticipated for the future, we decided to focus on lovastatin because of the availability of pharmacokinetics data, its safety profile, and its global consumption for cholesterol control.

Due to the fact that the mechanism of action for rottlerin is still debated and only limited data are available on pharmacology and toxicology, this compound has not yet been in clinical use. Furthermore, rottlerin was never approved by the FDA or other authorities for its use in patients, which in fact would have been one decisive criterion for our intention to reposition this drug as an MACC1 inhibitor. For the potential clinical translation of our findings, we therefore focused on lovastatin as a compound routinely used in the clinic and being more suited for repositioning in cancer therapy.

### Lovastatin decreases MACC1-associated migration in CRC cells

**Fig 4:**
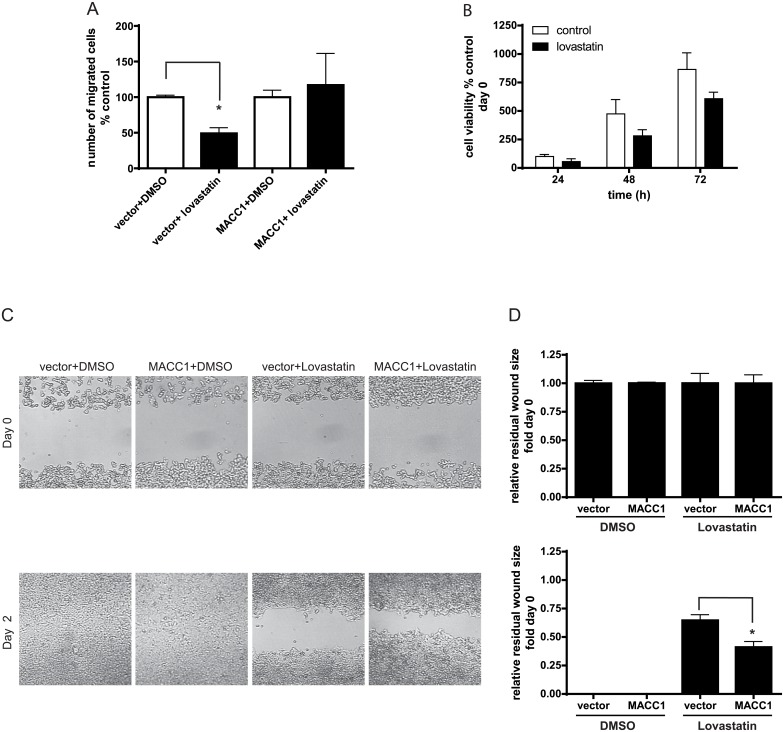
The effect of lovastatin on cell motility. (A) HCT116/vector and HCT116/MACC1 cells were treated with 5 μM lovastatin for 24 h, and migration was measured with the Boyden chamber assay. HCT116/MACC1 transfected with CMV promoter-driven MACC1 cDNA were used to revert the lovastatin effect. The migrated cells were stained with DAPI, and pictures were taken under a fluorescent microscope from 4 random fields per insert. The migrated cells were counted manually from those pictures and plotted in the bar graph. Data represent mean ± SEM (*n* = 2, *p* < 0.05). (B) The proliferation of lovastatin-treated versus nontreated cells was measured daily with MTT assays after the indicated time points. (C) The directed migration of lovastatin-treated HCT116 cells was analyzed by a wound healing assay. Microphotographs were taken 0 h, 24 h, and 48 h post treatment with 10x magnification. The assay was performed at least 2 times; 1 representative image is shown. (D) Wound size was analyzed using ImageJ and is expressed as the relative residual wound size compared to day 0. Control cells were treated with an equivalent amount of DMSO in all assays (*p* < 0.05).

A major phenotype imparted by MACC1 is increased migration of the CRC cells [1]. Thus, we investigated the effect of lovastatin on MACC1-mediated cell migration of HCT116/vector cells and HCT116/MACC1 using the Boyden chamber assay. HCT116/vector cells treated with 5 μM lovastatin for 24 h showed inhibition of cell migration to less than 50% of solvent-treated control cells (*p* < 0.05; Fig 4A). In contrast, HCT116/MACC1 cells with CMV promoter-driven MACC1 expression treated with lovastatin for 24 h showed no statistically significant inhibition of migration compared with the solvent-treated cells. As shown in Fig 4B, 5 μM lovastatin has no significant effect on proliferation/cell number in HCT116 cells at 24 h, suggesting that the effect on migration at this time point is mostly independent of cell proliferation.

We further analyzed the effect of lovastatin on directed migration in a wound healing assay. In the absence of lovastatin, HCT116/vector and HCT116/MACC1 cells entirely closed the inserted wound in 48 h (Fig 4C). Wound closure was impaired in lovastatin-treated HCT116/vector cells. In contrast, HCT116/MACC1 cells treated with lovastatin were able to infiltrate the wound and close the gap significantly faster compared to control cells, as shown by the representative figures and the corresponding quantification (Fig 4D; *p* < 0.05). This was also confirmed in DLD-1 cells (S2 Fig). In summary, treatment with lovastatin restricted cell motility. Ectopic overexpression of MACC1 was able to rescue lovastatin-mediated inhibition of cell motility, suggesting that the effect of lovastatin was specific to MACC1.

### Lovastatin interferes with the binding of transcription factors to the MACC1 promoter

**Fig 5:**
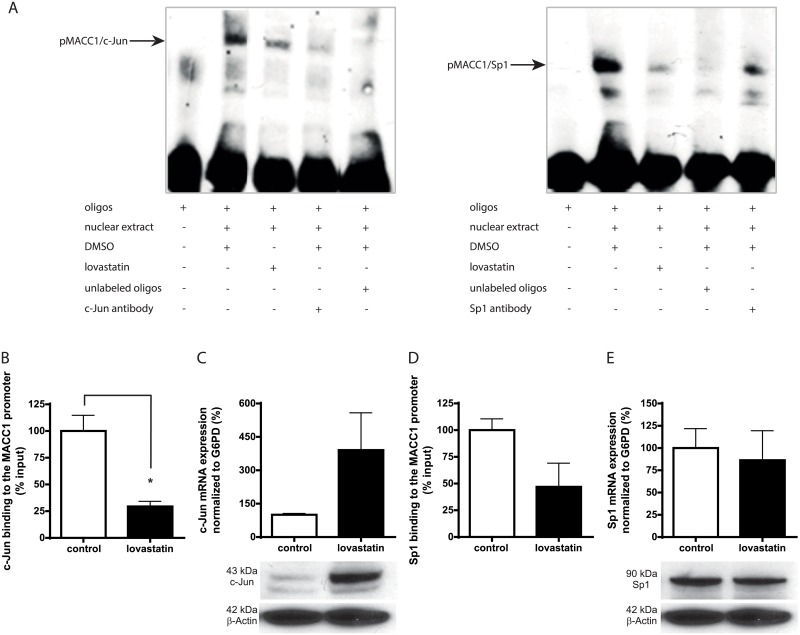
The effect of lovastatin on the binding of c-Jun and Sp1 to the MACC1 promoter. (A) An electrophoretic mobility shift assay (EMSA) was performed with equal amounts of nuclear extracts isolated from lovastatin- and solvent-treated cells. Lanes: (1) oligonucleotides only: 5′-biotin-labeled MACC1 promoter oligonucleotides flanking the binding sites specific for c-Jun (left) or Sp1 (right) without nuclear extract; (2) solvent control: nuclear extracts from solvent-treated HCT116 cells along with the respective labeled oligonucleotides; (3) lovastatin treatment: nuclear extracts from lovastatin-treated HCT116 cells along with the respective labeled oligonucleotides; (4) antibody: the DMSO-treated nuclear extracts were incubated with biotin-labeled oligonucleotides along with antibodies for c-Jun or Sp1; and (5) unlabeled oligos: a reaction with 100x molar excess of an unlabeled competitor sequence for indicating the specificity of the protein-DNA complexes; EMSA experiments have been performed at least 2 times, and a representative figure from each is shown. (B, D) Equal amounts of chromatin from solvent- and lovastatin-treated HCT116 cells were immunoprecipitated with antibodies for c-Jun (*p* < 0.05) (B) and Sp1 (D) and were quantified by quantitative real-time reverse-transcription polymerase chain reaction (qRT-PCR), using MACC1 promoter primers. The results were plotted as the percentage of input. (C, E) HCT116 cells were treated with 5 μM lovastatin for 24 h. Both mRNA expression and protein expression of c-Jun (C) and Sp1 (E) were analyzed. mRNA expression was normalized with G6PD and expressed as the percentage of the solvent-treated cells. β-actin was used as the loading control for western blotting. Data represent mean ± SEM (*n* = 2), *p < 0.05.

In our previous study, we described that c-Jun and Sp1 regulate MACC1 transcription by binding to its core promoter region [27]. Here, we identified that lovastatin inhibits MACC1 transcription. Therefore, we wanted to know if lovastatin influences c-Jun and Sp1 binding to the human MACC1 promoter. This was investigated by electrophoretic mobility shift assay (EMSA), using biotinylated oligonucleotides encompassing the c-Jun or Sp1 binding site of the MACC1 promoter as described earlier [27]. In solvent-treated HCT116 cells, a strong signal was observed because of the binding of c-Jun and Sp1 with their respective biotinylated oligonucleotides, consistent with our previous findings. However, treatment of HCT116 cells with lovastatin interrupted the binding of c-Jun and Sp1 with the MACC1 promoter (Fig 5A). The specificity of the c-Jun/MACC1 promoter complex was verified by the addition of unlabeled oligonucleotides and c-Jun antibody, both leading to the reduction in the signal (Fig 5A).

Similar results were found with the chromatin immunoprecipitation (ChIP) assay (Fig 5B and 5D). Solvent-treated cell extracts showed 3.3-fold and 2-fold higher enrichment of MACC1 promoter sequence after immunoprecipitation with c-Jun and Sp1 antibody, respectively, as compared with lovastatin-treated cell extracts. We further analyzed the expression levels of c-Jun and Sp1in lovastatin-treated cells, showing that drug treatment did not alter total Sp1 expression at mRNA and protein levels (Fig 5E). In contrast, c-Jun expression was found to be elevated at mRNA and protein levels upon lovastatin treatment (Fig 5C). Therefore, it can be deduced that the decrease in c-Jun and Sp1 binding to the MACC1 promoter is not attributable to the decrease in the c-Jun and Sp1 levels, and lovastatin is not an inhibitor of c-Jun or Sp1 expression. These results indicate that the small molecule lovastatin restricts c-Jun/MACC1 and Sp1/MACC1 promoter binding and thereby influences MACC1 gene transcription.

### In silico mode of action prediction for mevastatin, lovastatin, and rottlerin

**Fig 6:**
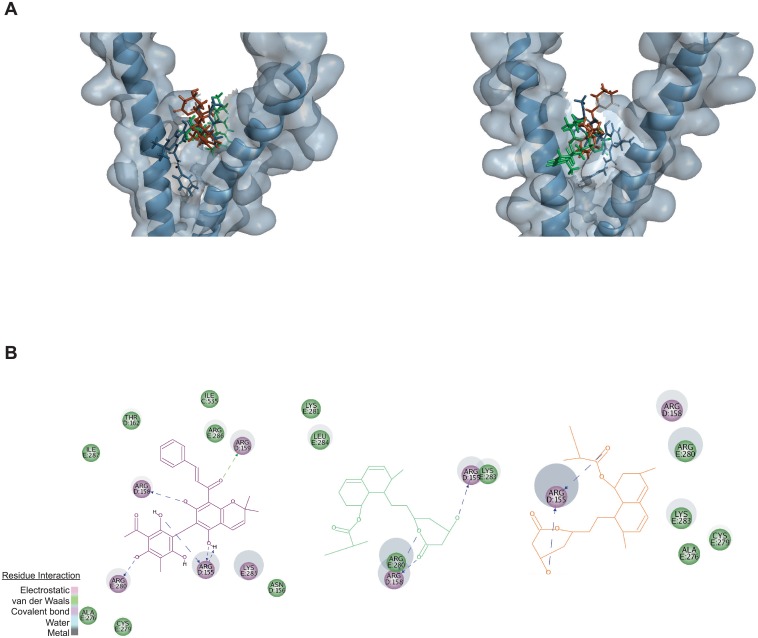
Molecular docking of transcriptional inhibitors to predict their mode of action. (A) 3D visualization of predicted drug binding to the DNA-binding domain (leucine zipper) of AP-1, viewed from different angles; mauve, rottlerin; green, mevastatin; and orange, lovastatin. (B) 2D visualization of predicted molecular interactions of the drugs with specific amino acid residues; the type of interaction is indicated by the color bar; mauve, rottlerin; green, mevastatin; and orange, lovastatin.

The reduction of MACC1-mRNA expression was verified by decreased binding of transcription factor complexes at AP-1/c-Jun-specific oligonucleotides of the MACC1 promoter. We then hypothesized the loss of DNA binding by competitive interaction of the drugs with the DNA binding site of AP-1. Therefore, we docked the drugs onto a published crystal structure of the leucine zipper of AP-1 (Fig 6A) and determined a docking score for each molecule. The docking score for rottlerin was 75.7, and the scores for lovastatin and mevastatin were 50.9 and 50.7, respectively. The molecular docking predicts interactions of all drugs with ARG155, ARG158, and LYS283, spanning both arms of the leucine zipper (Fig 6B). Since no cocrystallized ligand of this area other than DNA was available, we used published inhibitors of AP-1 to rate the docking scores and also docked them into the defined binding site. We found 4 substances, published by Chen et al., that reduced AP-1/DNA binding more than 90% at a concentration of 0.1 mg/ml [32]. The docking scores of shimobashiric acid C, salvianolic acid L, Mena987, and rosmarinic acid were predicted as 96.8, 82.2, 66.8, and 59.8, respectively.

### Lovastatin inhibits metastasis formation in mice

**Fig 7:**
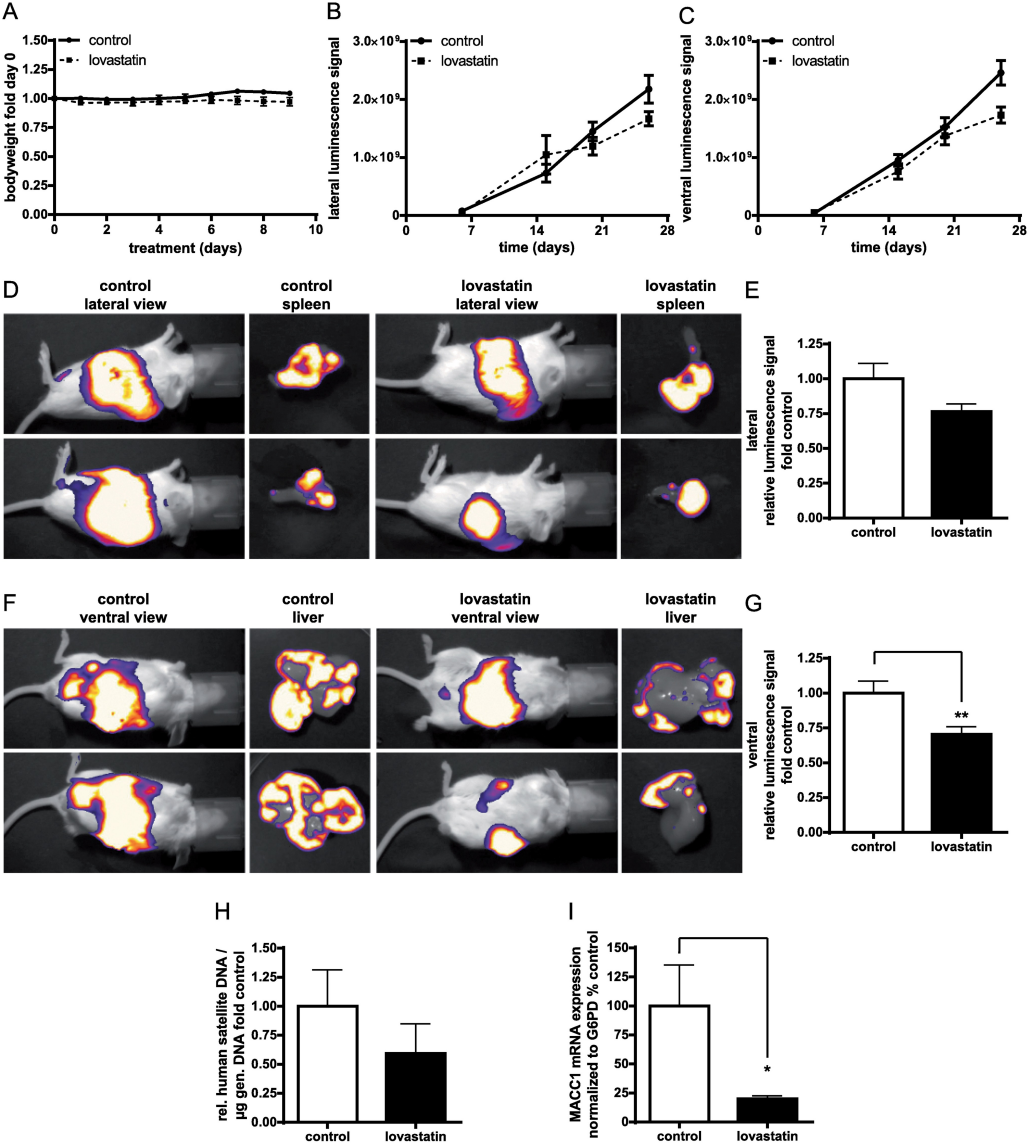
The effect of lovastatin on metastasis in mice. Severe combined immunodeficiency (SCID)-beige mice were intrasplenically transplanted with HCT116-CMVp-Luc cells and treated orally with 100 mg/kg lovastatin daily. Bioluminescence was measured by an intraperitoneal application of 150 mg/kg D-Luciferin and a sequence exposure of 20 s. (A) Acute toxicity was assessed in healthy animals treated with 100 mg/kg lovastatin. Body weight was measured daily for 10 d and is shown relative to day 0. (B, C) The lateral (B) and ventral signals (C) from tumors and metastases were monitored via bioluminescence imaging and quantified over time in solvent- and lovastatin-treated mice. (D, F) Representative pictures showing in vivo and ex vivo imaging of isolated organs from each group on day 26. All images are overlaid with the corresponding bright field pictures. Quantification of lateral (E) and ventral (*p* < 0.01) (G) signals was performed on day 26. All quantifications were performed using ImageJ software. (H) Human satellite DNA was quantified using quantitative polymerase chain reaction (qPCR) with equivalent amounts of genomic DNA obtained from the liver of each mouse. (I) MACC1 mRNA levels were determined from the livers using quantitative real-time reverse-transcription polymerase chain reaction (qRT-PCR) and normalized to human G6PD (*p* < 0.05). Data represent mean ± SE (*n* = 9 animals/group), *p < 0.05, **p < 0.01.

In order to analyze the effect of lovastatin on tumor growth and metastasis formation in mice, we first determined the tolerable doses of the drug. We chose an oral application route for a concentration range of 10 mg/kg up to 100 mg/kg lovastatin. Since we observed no toxicities (as a measure of body weight and general health condition) (Fig 7A), we finally selected a concentration of 100 mg/kg, which has been described to be safe and tolerable for the animals in a previous study [33], for further in vivo studies.

We next monitored the effect of lovastatin on metastasis formation over time in a mouse xenograft model by noninvasive in vivo bioluminescence imaging. Severe combined immunodeficiency (SCID)-beige mice were intrasplenically xenotransplanted with HCT116-CMVp-Luc cells and were randomized in 2 groups of *n* = 9 animals each. The bioluminescence measurement started at day 6 post intrasplenic transplantation of HCT116-CMVp-Luc cells. Lateral signals (representing the spleen as the site of the primary tumor) were observed in solvent- and lovastatin-treated mice and were assigned to the spleen. Ventral signals (representing the liver as the site of metastasis) were assigned to the liver, representing metastasis formation. Signals from both lateral and ventral imaging over the period of 26 d were quantified as shown in Fig 7B and 7C. On day 26, the animals in the solvent-treated control group started to show clear signs of morbidity due to tumor burden, which was not observed in the lovastatin-treated group. Thus, the experiment had to be terminated because of ethical reasons. Representative images indicating spleen and liver signals in vivo and ex vivo on day 26 are shown in Fig 7D and 7F. Quantification of the luminescence signal from the ventral side on day 26 showed a significant inhibition of liver metastasis under lovastatin treatment (*p* < 0.01), with a slight but not significant decrease in tumor growth in the spleen compared to the solvent-treated group (Fig 7E and 7G). We next analyzed the presence of human satellite DNA in the liver of the control versus the lovastatin-treated mice as a molecular marker for the appearance of metastases (Fig 7H). This analysis revealed that the livers from the lovastatin-treated group carried 40% less satellite DNA as compared to treated animals, supporting our bioluminescence finding from Fig 7C, 7F and 7G. In addition, MACC1 mRNA levels were quantified by quantitative real-time reverse-transcription polymerase chain reaction (qRT-PCR) to verify lovastatin-mediated transcriptional inhibition of MACC1 in vivo (Fig 7I). Lovastatin-treated animals showed significantly reduced MACC1 mRNA expression (*p* < 0.05), confirming that the drug acts as a transcriptional inhibitor of MACC1 and thus inhibits MACC1-induced metastasis formation in vivo. In order to analyze further drug-induced effects, we determined the mRNA expression of genes that can be regulated by lovastatin (DNMT1, Col1A1, and MCM2) [34–36]. DNMT1 and MCM2 showed no significant expression regulation upon treatment with lovastatin, whereas Col1A1 was significantly down-regulated, and HMGCR was significantly up-regulated (S3 Fig).

### Rottlerin inhibits metastasis formation in mice

**Fig 8:**
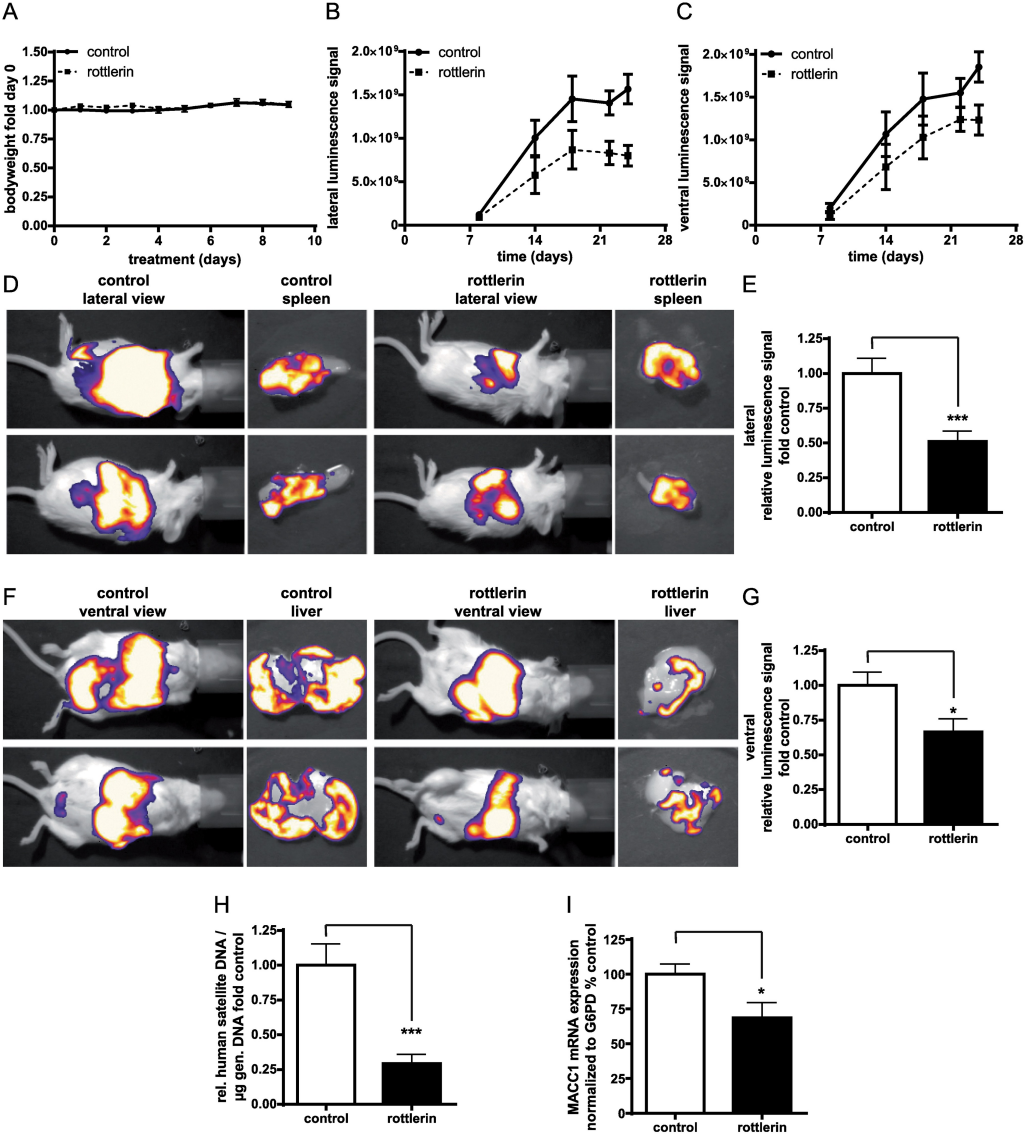
The effect of rottlerin on metastasis in the mice. Severe combined immunodeficiency (SCID)-beige mice were intrasplenically transplanted with HCT116-CMVp-Luc cells and treated orally with 100 mg/kg rottlerin daily. Bioluminescence was measured by an intraperitoneal application of 150 mg/kg D-Luciferin and a sequence exposure of 20 s. (A) Acute toxicity was assessed in healthy animals treated with 100 mg/kg rottlerin. Body weight was measured daily for 10 d and is shown relative to day 0. (B, C) The lateral (B) and ventral signals (C) from tumors and metastases were monitored via imaging and quantified over time in solvent- and rottlerin-treated mice. (D, F) Representative pictures showing in vivo and ex vivo imaging of mice and isolated organs from each group on day 24. All images are overlaid with the corresponding bright field pictures. Quantification of the lateral (*p* < 0.001) (E) and ventral (*p* < 0.05) (G) signals was performed on day 24. All quantifications are performed using ImageJ software. (H) Human satellite DNA was quantified using quantitative polymerase chain reaction (qPCR) with equivalent amounts of genomic DNA obtained from the liver of each mouse (*p* < 0.001). (I) MACC1 mRNA levels were determined from the livers using quantitative real-time reverse-transcription polymerase chain reaction (qRT-PCR) and normalized to human G6PD (*p* < 0.05). Data represent mean ± SE (*n* = 9 animals/group), *p < 0.05, ***p < 0.001.

As mentioned earlier, rottlerin was among the selected candidates for MACC1 inhibition that emerged among the most promising drugs from the HTS. Due to this fact, we were interested to validate if this drug is able to fulfill its proposed MACC1 inhibitory potential in vivo, which we consider as a decisive criterion for the HTS verification. Therefore, similar to the in vivo lovastatin study, we evaluated the effects of rottlerin as the second most promising drug that emerged from the HTS on MACC1-induced metastasis in mice. A previous study with rottlerin has used 0.012% rottlerin in food, corresponding to 25 mg rottlerin per kg bodyweight [37]. Herein, we employed an oral application route for a concentration range of 10 mg/kg up to 100 mg/kg rottlerin to determine tolerable doses. An oral concentration of 100 mg/kg was used for the in vivo studies, which was identified to be safe in the drug tolerability studies (Fig 8A). The bioluminescence measurement started at day 8 post intrasplenic transplantation of HCT116-CMVp-Luc cells. Signals from both lateral and ventral imaging over 24 d were quantified as shown in Fig 8B and 8C, demonstrating that rottlerin-treated animals had restricted primary tumor growth and reduced liver metastasis. Representative images indicating the lateral and ventral signals on day 24 are shown in Fig 8D and 8F. On day 24, the animals in the solvent-treated control group started to show clear signs of morbidity due to tumor burden, which was not observed in the rottlerin-treated group. Thus, the experiment was terminated. Endpoint quantification of lateral and ventral signals showed that the application of rottlerin reduced tumor growth (*p* < 0.001) and metastasis formation (*p* < 0.05) significantly (Fig 8E and 8G). We next analyzed the presence of human satellite DNA in the livers of the control versus the treated mice (Fig 8H). Our results validated that livers from the solvent-treated group had much more human satellite DNA as compared to the rottlerin-treated animals (*p* < 0.01), confirming the restricted metastatic ability of rottlerin-treated cells. Finally, MACC1 mRNA levels were quantified (Fig 8I). Rottlerin-treated animals showed significantly reduced MACC1 expression (*p* < 0.05), confirming that rottlerin acts as a transcriptional inhibitor of MACC1 and thus inhibits MACC1-induced metastasis formation in vivo. We analyzed further rottlerin-induced effects by determining the mRNA expression levels of genes that can be regulated by rottlerin (CDC20, mTor, and SKP2) [38–40]. No significant expression regulation upon treatment with rottlerin was observed (S3 Fig).

## Discussion

MACC1 has been established by numerous studies as a biomarker for prognosis of metastasis formation, survival, and prediction of therapy response in a broad variety of solid cancer types, such as in cancers of the gastrointestinal tract (e.g., CRC and esophageal, gastric, and pancreatic cancer) and hepatocellular, hepatobiliary, renal, bladder, breast, ovarian, cervical, lung, nasopharyngeal, salivary gland, and tongue cancer, as well as in glioblastomas and osteosarcomas. MACC1 acts as a key driver in tumor progression towards more advanced tumor stages and metastasis formation [2,24–26].

Here, we hypothesized the potential of MACC1 for targeted therapy and aimed to target MACC1 for intervention in CRC progression and metastasis formation. This is of particular interest since until now no inhibitors have been known for MACC1. Therefore, finding effective MACC1 inhibitors will add to the therapeutic possibilities for targeted intervention of tumor progression and metastasis progression. To achieve this, we employed HTS to identify inhibitors for MACC1 expression by using the human MACC1 promoter-driven luciferase reporter system. This HTS revealed mevastatin and rottlerin as the most promising hits for MACC1 transcriptional inhibition, providing the chance to reposition these drugs in cancer therapy.

Mevastatin (isolated from *Penicillium citrinum* and also known as compactin) was the first statin found to have a powerful inhibitory effect on HMG-CoA reductase [41–43]. In the present study, we demonstrated a novel target of this statin: MACC1. However, mevastatin was never approved by the FDA because of its lesser efficacy in patients and considerable toxicity in dogs. In contrast, lovastatin, a mevastatin analogue, was the first FDA-approved cholesterol-lowering drug. This drug has since then been long in clinical use, which makes lovastatin a valuable candidate for repositioning in cancer therapy [31].

From the HTS data, we decided to analyze the effects of mevastatin and lovastatin in more detail, demonstrating a concentration-dependent inhibition of MACC1 expression, subsequently restricting MACC1-induced cell motility in vitro. Lovastatin reduced MACC1 mRNA and protein expression in CRC cells, and the effects were dependent on drug concentration, time of treatment, and the cell line used. To the best of our knowledge, this is the first study demonstrating the effect of lovastatin on MACC1 expression. Consistent with our findings, lovastatin did not affect CMV promoter-driven MACC1 expression. Therefore, the effects we observed are specific for endogenous MACC1 regulated by the human MACC1 promoter. Moreover, our study demonstrates that lovastatin can restrict cell migration, thereby intervening in crucial metastatic capabilities. As statins target MACC1, which plays a decisive role in cellular motility, the effects of restricted migration could be at least partially mediated by reduced MACC1 levels. The rescue of the lovastatin-mediated effects on migration by ectopic CMV promoter-driven overexpression of MACC1 further strengthens the role of lovastatin in restricting MACC1-induced cell motility. However, the possibility of other lovastatin targets contributing to reduction in the aggressive phenotype still prevails. For instance, statins have already been described in the literature as anticancer agents acting via p38MAPK-p53-survivin signaling cascade or via the bone morphogenetic protein (BMP) pathway [44–46] or Rho signaling [47].

To understand further the impact of lovastatin on MACC1 transcriptional inhibition, we analyzed the binding of c-Jun and Sp1 to the human MACC1 promoter. C-Jun and Sp1 have been established in our previous study as crucial transcription factors that bind to the MACC1 promoter and regulate its expression [27]. Analysis of c-Jun and Sp1 binding with the MACC1 promoter revealed that lovastatin disrupted the binding of these transcription factors with the MACC1 promoter. This could be potentially due to several reasons like inactivation of their DNA binding domain, inhibition of accessory proteins required by these transcription factors to bind to the MACC1 promoter, inhibition of phosphorylation of these transcription factors, or reduced localization of these transcription factors in the nucleus, which remains unexplored. However, within the scope of this study, we show that lovastatin interfered with the transcription factor binding to the MACC1 promoter and thereby impairing MACC1 transcription in CRC cells, without inhibiting the expression of these transcription factors.

As mentioned above, inhibitors of transcriptional activity can act at many points in this process. To evaluate the mode of action of lovastatin, mevastatin, and rottlerin, which results in decreased binding of AP-1 to its specific binding site in the MACC1 promoter, we focused on a possible perturbation of the DNA-binding leucine zipper of AP-1, as previously published [48]. By molecular docking of the identified transcriptional inhibitors of MACC1, we found docking scores similar to substances that were screened to strongly inhibit AP-1/DNA binding activity [32]. Interestingly, the individual docking scores for the latter compounds inversely correlate with their published half maximal inhibitory concentrations (1.3 μM, 1.6 μM, 11.9 μM, and 16.2 μM, respectively) [32], indicating a relationship of docking score to inhibitory function, which can be extended also to the here reported transcriptional inhibitors of MACC1.

Furthermore, we analyzed the role of lovastatin as an inhibitor of MACC1-associated CRC progression and metastasis in mice. Our results demonstrate that lovastatin inhibits MACC1 expression at the site of the primary tumor in the spleen and intervenes with metastasis formation to the liver in the CRC xenografted SCID-beige mouse model. Thus, lovastatin application was effective to restrict cancer progression and metastasis formation in vivo. Recently, inhibition of tumor metastasis and growth by application of statins was also shown for metastatic melanoma via suppression of Rho signaling pathways [47].

Remarkably, in the molecular epidemiology of CRC study, including 1,953 patients with CRC and 2,015 controls, statin use was associated with a 47% decrease in the relative risk of developing CRC (odds ratio, 0.50) [49,50]. Very recently, statin use was associated inversely with the risk of CRC in a large cohort of patients with inflammatory bowel disease (odds ratio, 0.42) [51]. There have been numerous studies suggesting that statins can prevent CRC and prolong patient survival [47,49,52]. They may have an impact on the metastatic properties of CRC or may sensitize tumor cells to chemotherapeutic agents [53]. Despite all these studies, statins have failed to show improved outcome in clinical trials for CRC patients [54,55]. However, since almost 50% of CRC patients develop metachronous metastases, patient stratification based on the prognostic biomarker MACC1 might offer new options for subsequent therapy [56]. Thus, targeting MACC1 with drugs such as lovastatin might be meaningful as a prevention therapy to reduce the risk of metastasis development. Since statins are quite well tolerated by patients, they are promising drug-repositioning candidates for long-term treatment, with potential for the prevention/reduction of drug-mediated tumor progression and metastasis.

Meanwhile, several other HMG-CoA reductase inhibitors of the statin family like simvastatin, pravastatin, fluvastatin, atorvastatin, cerivastatin, and rosuvastatin came into the focus [57]. Further studies with the new-generation statins are of great interest to find the most potent statin to restrict CRC progression via MACC1 expression inhibition. In the present study, we solely focused on lovastatin as an attractive candidate for drug repositioning in CRC therapy.

We are certainly aware of the fact that lovastatin does not act exclusively via inhibiting MACC1 transcription. However, we demonstrated that treating different CRC cells with lovastatin led to significant reduction of MACC1 expression in concert with a reduction of various biological, MACC1-driven functions—most importantly, restricted metastasis formation. However, genes reported as lovastatin targets such as DNMT1 and MCM2 were not significantly regulated by lovastatin, whereas Col1A1 was significantly down-regulated. By contrast, HMGCR was up-regulated by lovastatin [58–60]. Further, since statins can be applied for years (even decades with tolerable side effects), a long-term treatment might offer new possibilities for CRC patients. Thus, we provide a conclusive line of evidence that statins can be effectively repositioned as inhibitors of MACC1, providing a novel therapy for effective metastasis prevention/inhibition in CRC with a promising future in clinics.

In addition to lovastatin, HTS led to the identification of rottlerin as another potential candidate to inhibit MACC1 transcription. As proof of concept of HTS-based MACC1 inhibitor identification, we tested rottlerin in vitro and in vivo. We confirmed the ability of this drug for reduction of MACC1 promoter activity and expression in cell culture and metastasis restriction in xenografted mice. Unlike lovastatin, rottlerin treatment also affected primary tumor growth significantly in vivo. Although we demonstrated here that rottlerin is a MACC1 transcription inhibitor, rottlerin was shown to possess a wide range of other medicinal potential, including antitumor, antioxidant, antiamyloid, and antibacterial abilities, mitochondrial uncoupling, mTOR inhibition, and anti-inflammatory activities involving a plethora of cellular targets [61]. However, genes such as CDC20, SKP2, and mTOR were not found to be significantly modulated by rottlerin. Further, studies have shown that rottlerin has good systemic distribution and bioavailability and thus possesses a good potential for being an anticancer drug [29,62]. We confirmed here the good tolerability in vivo. Our data provide evidence for broadened use of rottlerin as an antimetastatic drug acting via another novel target, MACC1, strengthening its future as a chemotherapeutic drug.

Taken together, our study uncovers the first small-molecule inhibitors of MACC1-induced cancer progression and metastasis formation in vitro and in vivo, thereby suggesting their strong therapeutic relevance. This drug repurposing might benefit patients who are at high risk for shorter survival caused by MACC1-induced metastasis.

## Materials and methods

### Ethics statement

All experiments were performed in accordance with the United Kingdom Co-ordinated Committee on Cancer Research (UKCCCR) guidelines and approved by the responsible local authorities (State Office of Health and Social Affairs, Berlin, Germany), REG0289/13.

Mice were anesthetized with isoflurane gas or Ketamin/Xylanzine. Animals were killed by cervical dislocation under anesthesia.

### Cell lines and growth conditions

Human CRC cell lines HCT116, DLD-1, SW620, and SW48, all originally from the American Type Culture Collection, were grown in DMEM or RPMI 1640 medium (Thermo Fisher Scientific, Waltham, Massachusetts) supplemented with 10% fetal bovine serum (Thermo Fisher Scientific). All cells were maintained at 37°C in a humidified incubator with 5% CO_2_. All cells tested negative for mycoplasma, verified regularly using the MycoAlert Mycoplasma detection kit (Lonza, Basel, Switzerland). Authentication of the cell lines was performed by short tandem repeat (STR) genotyping at the Leibniz-Institute DSMZ (Braunschweig, Germany). STR genotypes were consistent with published genotypes for these cell lines.

### Transfection of human CRC cells

The plasmid pGL4.17 (Promega, Fitchburg, Wisconsin) carrying the human MACC1 promoter sequence (−18 to −992 bp upstream of the transcription start site) upstream of the luciferase reporter gene has been described in our previous study [27]. HCT116 cells were transfected with this construct and selected with neomycin (Thermo Fisher Scientific) to generate HCT116-MACC1p-Luc cells. Stable expression of the transgene was controlled regularly by Steady Glow Luciferase Assay System (Promega) according to the manufacturer’s instructions.

For ectopic CMV promoter-driven overexpression of MACC1, the pcDNA3.1/MACC1 plasmid was used [63]. HCT116 cells were transfected with this construct to generate HCT116/MACC1 cells or with the empty pcDNA3.1 vector to obtain control HCT116/vector cells. Additionally, a CMV promoter-driven luciferase reporter HCT116 cell line (HCT116-CMVp-Luc) was used as described previously [64]. All transfections were performed with TransIT-2020 (Mirus, Madison, Wisconsin) according to the manufacturer’s instructions.

### HTS

Four thousand HCT116 cells stably expressing the MACC1 promoter-driven luciferase gene (HCT116-MACC1p-Luc) were seeded into each well of a white 384-well plate (Corning, Corning, New York) using an automatic pipetting system (Tecan AG, Männedorf, Switzerland). For the HTS, a compound library consisting of 30,000 compounds from ChemBioNet, which also included 1,280 compounds of the LOPAC (Sigma-Aldrich, St. Louis, Missouri), was used. The ChemBioNet collection of commercially available compounds has been provided by the Leibniz-Institute for Molecular Pharmacology (FMP), in cooperation with the Max-Delbrück-Center for Molecular Medicine, the Helmholtz-Center for Infection Research, and the University of Konstanz. The design and selection of compounds is based on a maximum common substructure analysis of the World Drug Index (WDI) [30]. The collection is extended by the donations of academic chemists and natural product collection of about 20,000 compounds (Analyticon Discovery, Potsdam, Germany).

The HTS was carried out for 24 h at a concentration of 5 μM per compound. The luciferase signal was measured using a microplate reader (Tecan). In parallel, a selectivity screen to eliminate general luciferase inhibitors was carried out using HCT116-CMVp-Luc cells. Compounds showing best selectivity and efficacy of MACC1 promoter inhibition were further used for a concentration-response screen at a concentration range of 0.025 μM to 25 μM. All measurements were made in triplicate. The parameters used in the screening are described in S1 Table. The results were calculated as percent luciferase activity compared to the respective controls.

### Drug treatments in vitro and in vivo

Mevastatin, lovastatin, and rottlerin were obtained from Santa Cruz Biotechnology (Dallas, Texas) and stored at −20°C. All drugs were solubilized in dimethyl sulfoxide (DMSO) for in vitro applications. The stock solution of 10 mM was prepared fresh every 2 wk and stored in small aliquots at −20°C to avoid repeated freeze thawing. To exclude adverse effects caused by DMSO, control cells were always treated with an equal amount of the solvent.

In vivo, lovastatin hydroxyl acid (sodium salt; Biomol, Hamburg, Germany) and rottlerin were administered daily orally as a suspension in 10% Kolliphor EL (Sigma-Aldrich) and 0.9% NaCl using a gavage tube. Control mice were treated with the appropriate volume of solvent solution (10% Kolliphor EL, 0.9% NaCl). The in vivo experiments were terminated when the animals in the control group showed signs of increased suffering due to tumor/metastasis burden and liver damage like swollen abdomen (ascites formation), reduced activity, and reduced food uptake (ethical/humane endpoint) (S4 Fig).

### RNA extraction and qRT-PCR

For expression analyses, 3x10^5^ cells were seeded in a 6-well plate, and after drug treatment, total RNA was isolated using the Universal RNA Purification Kit (Roboklon, Berlin, Germany) according to manufacturer’s instructions. RNA was quantified (Nanodrop, Peqlab, Erlangen, Germany), and 50 ng of RNA was reverse transcribed with random hexamers in a reaction mix (10 mM MgCl_2_, 1x RT-buffer, 250 μM pooled dNTPs, 1 U/μl RNAse inhibitor, and 2.5 U/μl Moloney Murine Leukemia Virus reverse transcriptase; all from Thermo Fisher Scientific) at 42°C for 15 min, 99°C for 5 min, with subsequent cooling at 5°C for 5 min. The cDNA was amplified by quantitative polymerase chain reaction (qPCR) using SYBR Green dye chemistry and the LightCycler 480 (Roche Diagnostics, Mannheim, Germany) under the following PCR conditions: 95°C for 2 min followed by 45 cycles of 95°C for 7 s, 60°C for 10 s, and 72°C for 20 s using primers for MACC1 and G6PD as described previously [1]. The primers are specific for the respective human gene (S5 Fig). The same protocol for qPCR has been employed for RNA from shock-frozen tumor and liver tissue samples from animals. Human satellite DNA in the liver sections of the control and treated mice was determined as previously described [65]. Data analysis was performed with the LightCycler 480 Software release 1.5.0 SP3 (Roche Diagnostics). Mean values were calculated from duplicate qRT-PCR reactions. Each mean value of the expressed gene was normalized to the respective mean amount of the G6PD cDNA.

### Protein extraction and western blotting

For total protein extraction, 3x10^5^ cells were plated in 6-well plates. After drug treatment, the cells were lysed with RIPA buffer (50 mM Tris–HCl; pH 7.5, 150 mM NaCl, and 1% Nonidet P-40, supplemented with complete protease inhibitor tablets; Roche Diagnostics) for 30 min on ice. Protein concentration was quantified with Bicinchoninic Acid Protein Assay Reagent (Thermo Fisher Scientific), according to the manufacturer’s instructions. Lysates of equal protein concentration were separated with sodium dodecyl sulfate-polyacrylamide gel electrophoresis (SDS-PAGE) and transferred to Hybond C Extra nitrocellulose membranes (GE Healthcare, Munich, Germany). Membranes were blocked for 1 h at room temperature with 5% nonfat dry milk in TBST buffer (10 mM Tris-HCl; pH 8, 0.1% Tween 20, and 150 mM NaCl). Membranes were then incubated overnight at 4°C with MACC1 antibody (Sigma-Aldrich, dilution 1:1000) or β-actin antibody (Sigma-Aldrich, dilution 1:10,000), followed by incubation for 1 h at room temperature with HRP-conjugated anti-rabbit IgG (Promega, dilution 1:10,000) or anti-mouse IgG (Thermo Fisher Scientific, dilution 1:10,000). Antibody-protein complexes were visualized with WesternBright ECL HRP substrate (Advansta, Menlo Park, California) and subsequent exposure to CL-Xposure Films (Thermo Fisher Scientific). Immunoblotting for β-actin served as the protein loading control.

### Transwell migration assay

HCT116 cells were first serum starved overnight. Then, 3x10^5^ serum starved cells in 300 μl of drug containing DMEM with 2% FBS were seeded into presoaked transwell chambers with a pore size of 8 μm (Corning). The control cells were treated with solvent. Six hundred and fifty μl of fresh medium with 10% FBS and drug was added to the bottom chamber. The cells that had migrated to the lower chamber were stained with DAPI, and 4 random pictures per transwell were taken under a fluorescent microscope (Axio Observer.Z1, Zeiss, Jena, Germany). The migrated cells were counted manually from those pictures. Results are expressed as the percent number of migrated cells compared to controls.

### Wound healing assay

The wound healing assay was used to analyze directed cell migration. On day 0, 5x10^4^ cells were seeded into cell culture inserts (ibidi, Martinsried, Germany) to create a wound. After an attachment time of 24 h, the culture inserts were gently removed, and a wound of about 500 μm width was inflicted. Subsequently, medium containing the drug or solvent was added. The progress of wound closure was monitored daily, and microphotographs of 10x and 40x magnification were taken with the Leica DM IL light microscope (Leica Microsystems, Heerbrugg, Switzerland) from day 0 up to day 2. Results were quantified using ImageJ 1.48v (NIH, Bethesda, Maryland) and the MRI wound healing tool (available online) as relative residual wound size compared to starting day 0. Each wound healing assay was performed in duplicate.

### MTT cell viability assay

For determination of cell viability and proliferation, 4x10^3^ cells were plated into 96-well plates and were allowed to accommodate for 24 h before treatment was started. Cells were treated daily for 4 d with inhibitor or solvent. For determination of viable cells, 3-(4,5-dimethylthiazol-2-yl)-2, 5-diphenyltetrazolium bromide (MTT; Sigma-Aldrich) was added to a final concentration of 0.5 mg/ml and incubated for 3 h at 37°C in a humidified incubator. MTT was reduced to purple formazan crystals by the mitochondria of living cells, and the decrease in metabolized MTT represented decreased cell viability and number. Formazan crystals were dissolved in 150 μl of DMSO, and the absorption was measured at 560 nm in the multiwell reader (Tecan infinite 200 PRO, Tecan). Each cell proliferation experiment was performed in triplicate. Results are expressed as percent viable cells compared to solvent-treated controls.

### ChIP

A ChIP assay was performed using EZ-Magna ChIP kit (Merck, Darmstadt, Germany) as per the manufacturer’s instruction. Cells (2x10^6^) were plated in 10-cm dishes. Twenty-four hours after drug treatment, the cells were cross-linked with 1% formaldehyde for 10 min at room temperature, lysed in the lysis buffer provided in the kit, and sonicated for 25 pulses at 40% output to release chromatin. Cell lysates were then centrifuged at 10,000 rpm for 10 min, and supernatant was collected in a new tube. One percent of this solution was stored at 4°C until the elution step and served as an input control. The protein-DNA complexes were precipitated on addition of polyclonal antibodies for c-Jun or Sp1 (Cell signaling, Danvers, Massachusetts) to the chromatin solution and incubated overnight at 4°C. Magnetic beads were then added and incubated for another 2 h at 4°C. Nonbound protein was removed by washing twice with the Wash Buffers provided in the kit. The protein-DNA complex was eluted from the beads with the elution buffer, followed by centrifugation at 3,000 rpm for 1 min. Cross-linking of protein and DNA was reversed at 68°C overnight, and residual protein was digested by proteinase K at 55°C for 2 h. DNA was purified by column purification. The extracted DNA was subjected to quantitative PCR as described above with the MACC1 promoter primers described before [27].

### EMSA

EMSA was performed using the LightShift Chemiluminescent EMSA Kit (Thermo Fisher Scientific) as per the manufacturer’s instruction. Briefly, 2x10^6^ cells were seeded in a 10-cm culture dish and incubated for 24 h for cell adherence. Twenty-four hours after drug or solvent treatment, nuclear extracts were prepared using NE-PER nuclear and cytoplasmic extraction reagent (Thermo Fisher Scientific) as per the manufacturer’s protocol. As described earlier, 5′-labeled biotin oligonucleotides for the putative binding sites for c-Jun and Sp1 in the human MACC1 promoter were used [27]. In a total volume of 20 μl, 5 μl of nuclear extracted protein was incubated for 30 min at room temperature along with 0.05% w/v poly dI·dC, 0.5 mM Tris, 0.05 mM EDTA, 2.5% v/v glycerol, 0.2% v/v NP-40, 5 mM MgCl_2,_ and double-stranded biotinylated oligonucleotides containing the respective transcription factor binding site as present in the MACC1 promoter. For the super shift assay, 5 μg c-Jun or Sp1 antibody was added before the addition of the corresponding oligonucleotide and incubated for 30 min on ice, whereas 100-fold molar excess of unlabeled oligonucleotides was used in the competition experiments. Electrophoretic separation of the protein-oligonucleotide complexes was performed by precast Novex 6% TBE gels (Thermo Fisher Scientific) in TBE buffer (45 mM Tris, 45 mM boric acid, 1 mM EDTA, pH 8.3) for 60 min at 100 V. Capillary transfer of the protein-oligonucleotide complexes to the Hybond-N nylon membrane (GE Healthcare) was performed in 20x SSC buffer (3 M NaCl, 300 mM Na_3_C_6_H_5_O_7_, pH 7) overnight. Transferred DNA was cross-linked to the membrane at 250 mJ/cm^2^ for 1 min in the FL-20-M FluoLinkCrosslinker (Bachofer, Reutlingen, Germany). Visualization of biotin-labeled DNA was performed with the LightShift Chemiluminescent EMSA Kit (Thermo Fisher Scientific) according to the manufacturer’s instructions.

### Molecular docking

Searching in the protein database (PDB) [66] for the transcription factor AP-1 results in 4 crystal structures and 1 NMR structure. To evaluate which structure might be most suitable for docking studies, a superposition of these 5 PDB entries was performed using PyMOL, an open-source and widely used biomolecular visualization program [67]. Based on the resulting superpositions, we decided to use the PDB structure 1S9K for further evaluation.

To prepare the small molecules for docking, Discovery studio 4.1 was used [68]. This process comprises adding hydrogens, normalizing the ionization state, generating possible tautomers, fixing valencies, and generating three-dimensional coordinates. Additionally, a minimization step was applied to generate low-energy conformers, by applying the Smart minimizer. Docking studies were carried out by using GOLD suite 5.2 [69] with the Goldscore scoring function, where the integrated wizard was used to set up and run the docking. The first step comprises the preparation of the protein. Therefore, hydrogens were added, water and possible side chain rotamers were removed, and the cocrystallized DNA was excluded. We focused on a possible inhibition of the AP-1/DNA interaction to identify any drug binding site and defined a radius of 12 angstrom around the AP-1/DNA interacting atoms to cover the complete binding area. Additional options, like protein and ligand flexibility, were kept in default configuration.

### In vivo bioluminescence imaging of tumor growth and metastasis formation

All experiments were performed in accordance with the UKCCCR guidelines and approved by the responsible local authorities (State Office of Health and Social Affairs, Berlin, Germany).

HCT116-CMVp-Luc cells (3x10^6^ cells/animal) were intrasplenically transplanted into 6-wk-old female SCID-beige mice (Charles River, Wilmington, Massachusetts). SCID-beige mice were randomly assigned 24 h after cell transplantation to 2 groups of 9 animals each. Mice were then treated orally with daily doses of either solvent (10% Kolliphor in 0.9% NaCl) or 100 mg/kg body weight of lovastatin hydroxyl acid or rottlerin for up to 4 wk. Tumor growth and metastasis formation were monitored by bioluminescence imaging using the NightOWL LB 981 imaging system (Berthold Technologies, Bad Wildbad, Germany). For bioluminescence imaging, mice were anesthetized with isoflurane gas and received intraperitoneally 150 mg/kg D-luciferin (Biosynth, Staad, Switzerland). Tumor growth and metastasis formation were imaged and quantified by WinLight (Berthold Technologies) and ImageJ 1.48v. The experimental endpoint was defined by ethical guidelines of animal care (S4 Fig). After the animals were killed, the spleen (the tumor implantation site) and the liver (the metastasis target organ) were removed and shock frozen in liquid nitrogen, and cryosections were performed for isolation of genomic DNA and total RNA (DNA/RNA/Protein extraction kit, Roboklon) for further analyses.

### Statistical analysis

All calculations and statistical analyses were performed with GraphPad Prism version 5.01. Comparison of the 2 groups was done by an unpaired *t* test. Comparison of a control versus multiple respective treated groups was performed by one-way analysis of variance (*ANOVA*) Dunnett’s multiple comparison test. All significance tests were 2-sided, and *p*-values less than 0.05 were defined as statistically significant.

## Supporting information

S1 Fig Real time proliferation analysis for cytotoxicity effects of lovastatin and rottlerin. Cells were seeded at high density comparable to migration/invasion experiments 24h before drug treatment. Cells were treated with lovastatin (HCT116 5 μM, DLD1 30 μM, SW620 50 μM and SW48 2.5 μM) and rottlerin (HCT116 2.5 μM, DLD1 2.5 μM, SW620 20 μM and SW48 5 μM) and observed for additional 4 days. The experiment was done twice, each in triplicate.
(EPS)

S2 Fig Rescue of migratory phenotype *in vitro* in DLD-1 cells. The inhibition of directed migration in the scratch assay of DLD-1 cells was rescued with ectopic CMV promoter driven overexpression of MACC1. Cell growth was monitored in the IncuCyte instrument for 24 h. Wound closure is shown relative to medium treated wild type cells ± (n = 2 in quadruplicate). The experiment demonstrates rescue of the migratory phenotype by MACC1 overexpression.
(EPS)

S3 Fig Evaluation of further potential transcriptional target genes of lovastatin and rottlerin. Gene expression of Col1A1, DNMT1, MCM2, CDC20, mTor, SKP2, and HMGCR was analyzed by qRT-PCR after *in vivo* treatment with lovastatin and rottlerin.
(EPS)

S4 Fig Overview of liver damage at the endpoint of *in vivo* experiments. SCID-beige mice were intrasplenically transplanted with HCT116-CMVp-Luc cells and treated orally with (A) 100 mg/kg lovastatin or (B) rottlerin daily. Bioluminescence was measured to monitor tumor growth and metastasis formation over time. When the experiments had to be terminated due to tumor and metastasis load in the control group (ethical endpoint), the organs, especially liver and spleen, were carefully examined *ex vivo*. In the control group most animals showed strong liver damage due to excessive growth of metastases. This was leading to e.g. ascites formation and intra-abdominal bleedings. Therefore, the *in vivo* experiments had to be terminated at day 24 or 26 respectively to meet ethical criteria.
(EPS)

S5 Fig Specificity of MACC1 primers. The human MACC1 specific primers generate a standard curve using a serial dilution of plasmid DNA encoding human MACC1. The amplification curves (A, left panel, purple curves, B) shift upon dilution of DNA to the right. The same primers are not able to form a standard curve with the same amount of plasmid DNA encoding murine MACC1 (A left panel, red and green curves). Primers generated to detect murine MACC1 can form a standard curve with the correct DNA, but not with human DNA (A, amplification curves right panel, B). These data demonstrate the specificity of the human primers.
(EPS)

S1 Table High throughput screening parameters. High-throughput screening parameters.
(DOCX)

S2 Table Top 10 hits from high throughput screening. The top 10 hits from high-throughput screening.
(DOCX)

S1 Data Data for figures and supporting figures. All data used to create the figures in the manuscript.
(XLSX)

## Abbreviations

BMP: bone morphogenetic protein
ChIP: chromatin immunoprecipitation
CRC: colorectal cancer
EMSA: electrophoretic mobility shift assay
FDA: Food and Drug Administration
HTS: high-throughput screening
LOPAC: Library of Pharmacologically Active Compounds
MACC1: Metastasis Associated in Colon Cancer 1
qPCR: quantitative polymerase chain reaction
qRT-PCR: quantitative real-time reverse-transcription polymerase chain reaction
SCID: severe combined immunodeficiency
