# Supplementary material for: Statin and rottlerin small-molecule inhibitors restrict colon cancer progression and metastasis via MACC1
Source: PLoS Biol. 2017 Jun 1;15(6):e2000784. doi: 10.1371/journal.pbio.2000784 (PMC5453412; doi:10.1371/journal.pbio.2000784)
Supplement: S1 Table — High-throughput screening parameters. (DOCX) [file pbio.2000784.s006.docx]

**Table S1. High throughput screening parameters.**

| ***Category*** | ***Parameter*** | ***Description*** |
| --- | --- | --- |
| Assay | Type of assay | Cell based Luciferase Reporter assay |
|  | Target | MACC1 promoter |
|  | Primary measurement | Bioluminescence |
|  | Key reagents | Steady glow reagent from Promega |
|  | Assay protocol | 1. Day1: cell seeding 5,000 cells/well in 40 µl RPMI medium 2. Day2: add compounds 5 µM (Tecan Wall E, 0.2 µl, fc0.5% DMSO) 3. Day3:  - Aspirate 30 µl medium with Tecan/WallE - Add 10µl steady-Glo luciferase reagent, centrifuge, incubate 15 min - Measure luminescence at 500 ms integration time |
| Library | Library size | 30,000 compounds library |
|  | Library composition | Drug like molecules and LOPAC compounds |
|  | Source | ChemBioNet |
| Screen | Format | 384 well type |
|  | Concentration tested | 5 µM |
|  | Plate controls | HCT116-pGL4.17 empty vector cells,  and Untreated and DMSO treated HCT116-MACC1p-Luc cells |
|  | Reagent/ compound dispensing system compounds | 5 µM (TecanFreedom Evo with TeMO384 pipetting head , 0.2 µl, 0.5% DMSO) |
|  | Assay validation/QC | Z factor > 0.4 |
|  | Correction factors | 1.48 |
|  | Normalization | Normalized percent inhibition, Z score |
|  | Hit criteria | Z score< -3 |
| Post-HTS analysis | Additional assay(s) | Counter/specificity screen with HCT116-CMVp-Luc cells |
|  | Confirmation of hit purity and structure | Mass spectrometry |
