# Supplementary material for: Statin and rottlerin small-molecule inhibitors restrict colon cancer progression and metastasis via MACC1
Source: PLoS Biol. 2017 Jun 1;15(6):e2000784. doi: 10.1371/journal.pbio.2000784 (PMC5453412; doi:10.1371/journal.pbio.2000784)
Supplement: S2 Table — The top 10 hits from high-throughput screening. (DOCX) [file pbio.2000784.s007.docx]

**Table S2. Top 10 hits from High throughput screening.**

|  | Chemical formula | IUPAC name | IC_50_  (µM) |
| --- | --- | --- | --- |
| 1 | C_30_H_28_O_8_ | (E)-1-[6-[(3-acetyl-2,4,6-trihydroxy-5-methylphenyl)methyl]-5,7-dihydroxy-2,2-dimethylchromen-8-yl]-3-phenylprop-2-en-1-one **(Rottlerin)** | **1,06** |
| 2 | C_23_H_34_O_5_ | [(1S,7S,8S,8aR)-8-[2-[(2R,4R)-4-hydroxy-6-oxooxan-2-yl]ethyl]-7-methyl-1,2,3,7,8,8a-hexahydronaphthalen-1-yl] (2S)-2-methylbutanoate **(Mevastatin)** | **3,10** |
| 3 | C_27_H_26_N_4_O_3_S | 2,3-dihydroindol-1-yl-[1-[(2-phenyl-3H-benzimidazol-5-yl)sulfonyl]piperidin-3-yl]methanone | **1,98** |
| 4 | [C_26_H_3_0N_2_O_6_S](http://pubchem.ncbi.nlm.nih.gov/search/#collection=compounds&query_type=mf&query=C26H30N2O6S) | [2-(3,5-dimethoxyphenyl)-3-sulfanylidene-1,4-diazaspiro[4.5]dec-1-en-4-yl]-(3,4,5-trimethoxyphenyl)methanone | **1,98** |
| 5 | [C_22_H_18_N_2_O_5_S](http://pubchem.ncbi.nlm.nih.gov/search/#collection=compounds&query_type=mf&query=C22H18N2O5S) | N-(2,3-dihydro-1,4-benzodioxin-6-yl)-2-(5,5-dioxophenothiazin-10yl)acetamide | **0,04** |
| 6 | [C_24_H_19_F_3_N_6_O_2_S](http://pubchem.ncbi.nlm.nih.gov/search/#collection=compounds&query_type=mf&query=C26H30N2O6S) | 4-[2-[[3-[3-(trifluoromethyl)phenyl]triazolo[1,5-a]quinazolin-5-yl]amino]ethyl]benzenesulfonamide | **9,48** |
| 7 | [C_27_H_20_N_4_O_2_](http://pubchem.ncbi.nlm.nih.gov/search/#collection=compounds&query_type=mf&query=C27H20N4O2) | N-[4-(6-benzamido-1H-benzimidazol-2-yl)phenyl]benzamide | **1,59** |
| 8 | [C_18_H_9_N_3_O_3_](http://pubchem.ncbi.nlm.nih.gov/search/#collection=compounds&query_type=mf&query=C18H9N3O3) | 4-Nitro-benzo[de]benzo[4,5]imidazo[2,1-a]isoquinolin-7-one | **0,27** |
| 9 | [C_13_H_8_BrF_6_N_3_O_2_S](http://pubchem.ncbi.nlm.nih.gov/search/#collection=compounds&query_type=mf&query=C13H8BrF6N3O2S) | 2-amino-N-[3,5-bis(trifluoromethyl)phenyl]-5-bromopyridine-3-sulfonamide | **1,88** |
| 10 | [C_21_H_20_Cl_2_N_2_O_4_S](http://pubchem.ncbi.nlm.nih.gov/search/#collection=compounds&query_type=mf&query=C21H20Cl2N2O4S) | (4-(2,4-dichlorophenyl)-2,2-dimethyl-5-thioxo-2,5-dihydro-1H-imidazol-1-yl)(3,4,5-trimethoxyphenyl)methanone | **4,76** |
